# Rapid detection of NDM, KPC and OXA-48 carbapenemases directly from positive blood cultures using a new multiplex immunochromatographic assay

**DOI:** 10.1371/journal.pone.0204157

**Published:** 2018-09-14

**Authors:** Axel Hamprecht, Jörg Janne Vehreschild, Harald Seifert, Ahmad Saleh

**Affiliations:** 1 Institute for Medical Microbiology, Immunology and Hygiene, University Hospital of Cologne, Cologne, Germany; 2 DZIF (German Centre for Infection Research), partner site Bonn-Cologne, Germany; 3 Department I for Internal Medicine, University Hospital of Cologne, Cologne, Germany; University Medical Center Groningen, NETHERLANDS

## Abstract

Bloodstream infections caused by carbapenemase-producing Enterobacteriaceae (CPE) are associated with treatment failure and increased mortality. Detection of CPE from blood cultures (BC) by standard methods takes 16–72 hours, which can delay the initiation of appropriate antimicrobial therapy and compromise patient outcome. In the present study, we developed and evaluated a new method for the rapid detection of carbapenemases directly from positive BC using a new multiplex immunochromatographic test (ICT). The new ICT was assessed using 170 molecularly characterized *Enterobacteriaceae* clinical isolates including 126 CPE (OXA-48-like (N = 79), KPC (N = 18) and NDM (N = 29)). After spiking with bacteria and incubation in a BC system, blood from positive BC bottles was hemolyzed, bacteria concentrated by centrifugation and lysed. The lysate was transferred to the RESIST-3 O.K.N. ICT (Coris BioConcept, Gembloux, Belgium), which detects OXA-48-like, KPC and NDM carbapenemases. The final results of the ICT were read when they became positive, at the latest after 15 min. All CPE isolates (126/126) were correctly detected with the new protocol (100% sensitivity, 100% specificity). There was perfect concordance between ICT results and molecular characterization. Total time to result was 20–45 min.

Conclusions: This proof-of-principle study demonstrates that with the newly developed method, OXA-48-like, KPC and NDM carbapenemases can be reliably detected directly from positive BC bottles. The new method is more rapid than other currently available assays and can be performed in any routine microbiology laboratory. This can help to rapidly identify patients with CPE BSI and optimize the management of patients with these difficult-to-treat infections. Further studies are needed to assess the performance of the ICT in routine diagnostics.

## Introduction

With the increase of carbapenemase-producing Enterobacteriaceae (CPE), the treatment of severe bacterial infections has become more difficult, especially in high prevalence regions. Bloodstream infections (BSI) caused by CPE often delay appropriate antimicrobial therapy and are associated with an increased mortality [1]. The rapid detection of CPE as causative pathogens of BSI is therefore of paramount importance, as it has the potential to improve patient management and outcome. Among the different carbapenemases, OXA-48-like, KPC, VIM, IMP and NDM type carbapenemases are the most important worldwide [2].

The phenotypic detection of carbapenemases can be difficult by standard techniques. The detection of CPE from blood cultures in routine laboratories is lengthy and usually takes 16–72 hours after a blood culture is flagged positive (16–20 hours for direct susceptibility testing plus additional time for confirmatory tests). Reliable phenotypic tests (e.g. inhibitor based tests) or colorimetric tests (e.g. CarbaNP) require 2 to 24 hours after the first result of the susceptibility tests [3, 4]. Molecular detection of carbapenemases by PCR is considered the gold standard, but is costly, not performed in many laboratories, and/or available only during the main working hours [4].

Immunochromatographic lateral flow tests (ICT) have recently been developed for CPE detection from cultures on solid media. The ICTs detect epitopes specific for a carbapenemase, are very rapid (≤15 min) and have demonstrated excellent sensitivity and specificity across different carbapenemase variants and species [3, 5–7].

The RESIST-3 O.K.N. assay (Coris BioConcept, Gembloux, Belgium) is a new multiplex ICT based on monoclonal antibodies against carbapenemases of the type OXA-48-like, KPC and NDM. It has been evaluated for CPE detection from solid media [8, 9]. In the present study, we developed and evaluated two new protocols for the rapid detection of CPE directly from positive blood cultures using this ICT.

## Material and methods

### Ethics

The study was approved by the ethics committee of the University Hospital Cologne (approval number 08–160) and written informed consent was obtained from all healthy volunteers who donated blood for this study.

### Bacterial isolates

In total, 126 *Enterobacteriaceae* clinical isolates harboring different carbapenemases and 44 *Enterobacteriaceae* clinical isolates that were carbapenemase-negative were included in the analysis. *Klebsiella pneumoniae* was the most frequent species (N = 84), followed by *Escherichia coli* (N = 53) and *Enterobacter cloacae* (N = 15) as depicted in Table 1. All isolates were from the University Hospital Cologne or from previous studies and had been molecularly characterized as reported previously [3, 10–13]. Carbapenemase-producing isolates included the three carbapenemases OXA-48-like (N = 79), NDM (N = 29) or KPC (N = 18).

**Table 1:** Isolates included in the study.

	*K*. *pneumoniae*	*E*. *coli*	*E*. *cloacae*	*E*. *aerog*.	*C*. *freundii*	Other species	All isolates
**Carbapenemase** **positive**	73	33	9	2	4	5	126
OXA-48-like	44	26	4	2	3		79
OXA-48	30	17	3	2	2		54
OXA-162	3	2	1		1		7
OXA-181	4	4					8
OXA-204	1						1
OXA-232	5	2					7
OXA-244	1	1					2
KPC	16				1	1	18
KPC-2	15				1	1	17
KPC-3	1						1
NDM	12	5	5			4	26
NDM-1	12	3	5			4	24
NDM-5		1					1
NDM-7		1					1
OXA-48-like/NDM							3
NDM-1/OXA-48		1					1
NDM-5/OXA-181		1					1
NDM-1/OXA-232	1						1
							0
**Carbapenemase negative**	11	20	6	4	1	2	44
**Total**	84	53	15	6	5	7	170

*Klebsiella (K*.*) pneumoniae*, *Enterobacter (E*.*) cloacae*, *Enterobacter (E*.*) aerogenes*, *Citrobacter (C*.*) freundii*, other species: *Proteus mirabilis* (N = 2), *Enterobacter asburiae* (N = 1), *Citrobacter brakii* (N = 1), *Providencia stuartii* (N = 1), *Raoultella ornitholytica* (N = 1), *Serratia marcescens* (N = 1)

### Inoculation and processing of blood culture bottles

A bacterial suspension equivalent to 0.5 McFarland was diluted 1:1000. Subsequently 10 μL of this solution was mixed with 5 mL of human blood from healthy volunteers for a final inoculum of ~300 cfu/mL of blood and inoculated into a BD Bactec Plus Aerobic blood culture bottle (Becton Dickinson, Heidelberg, Germany). The bottles were then incubated in a Bactec FX automate (Becton Dickinson) until they flagged positive. All positive blood culture bottles were controlled for purity by inoculation of 100 μL blood culture fluid onto sheep blood agar.

Additionally, 10 NDM producing isolates were tested using BactAlert Aerobic Plus BC bottles (bioMérieux, Nürtingen, Germany). The procedure was identical to that using Bactec bottles, except that bottles were not incubated in an automated BC system but in a standard incubator until a color change was visualized at the bottom of the BC bottle.

### Immunochromatographic test from blood cultures

From positive bottles, different volumes of blood culture fluid was drawn and mixed with a hemolyzing agent at the ratio of 5:1. For hemolysis of erythrocytes, different substances were tested, including 5% saponin (Carl Roth, Karlsruhe, Germany), Triton-X (Sigma-Aldrich, Schnelldorf, Germany), ammonium-chloride/potassium hydrogen carbonate [0.15 M NH_4_Cl, 1 mM KHCO_3_] (Merck, Darmstadt, Germany), 10% SDS solution (Fa Gibco, Darmstadt, Germany). After hemolysis, the mixture was spun down in a table top centrifuge (13 000 g, 1 min). The sediment was washed with 1000 μL *aqua dest*. If the suspension was still red, it was washed a second time using 1000 μL phosphate buffered saline (PBS). Subsequently, the pellet was lysed with 10 drops of LY-A solution, a lysis solution provided with the test kit, and then 90 μL of the lysate was transferred to the lateral flow test. The final results of the ICT were read when they became positive, at the latest after 15 min.

**Fig 1:**
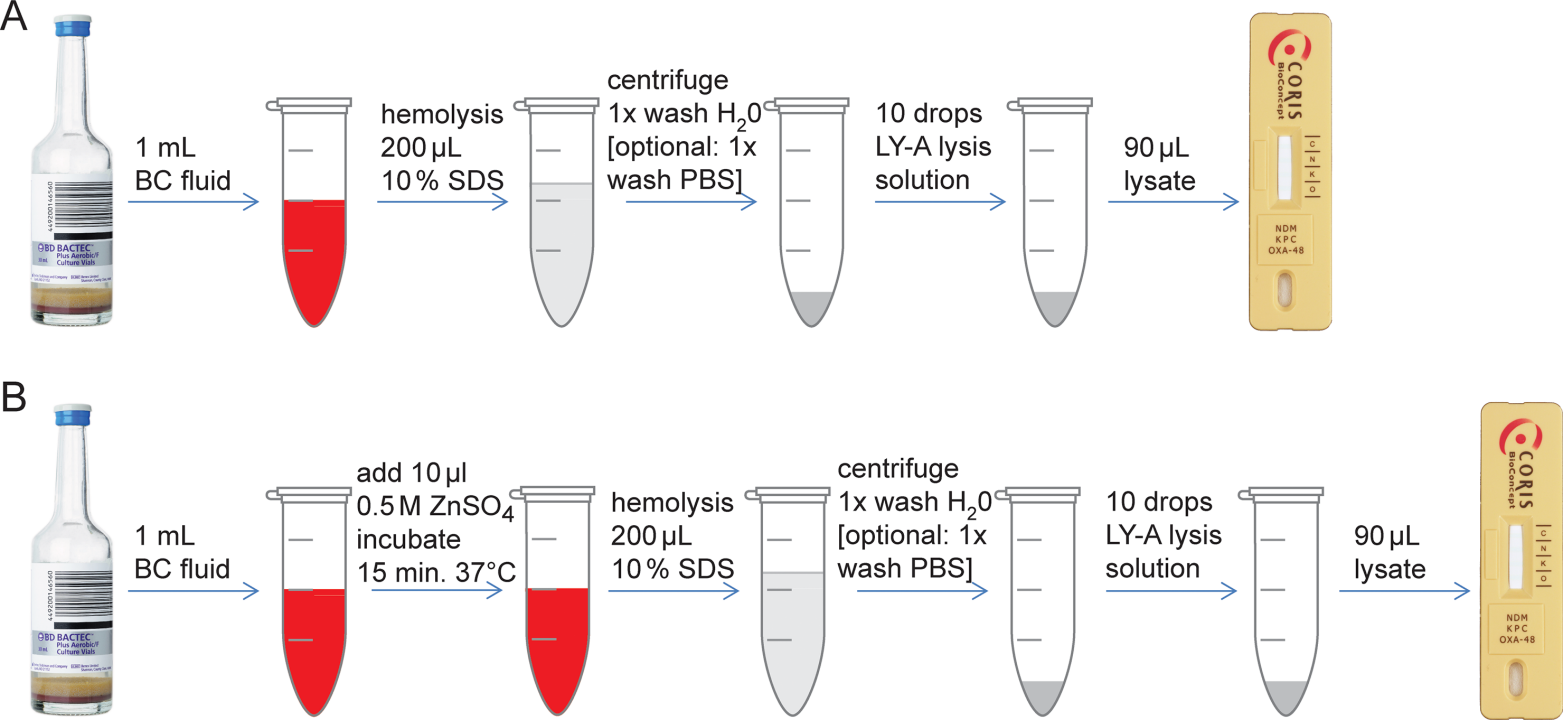
Workflow for the preparation of ICT directly from positive blood cultures. (A) Short protocol; (B) Advanced protocol for improved detection of NDM. SDS: sodium dodecyl sulfate; PBS: phosphate buffered saline.

A short protocol using 10% SDS is depicted in Fig 1.

**Fig 2:**
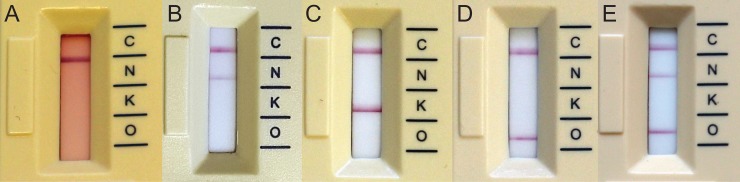
Result of the ICT. (A) no pretreatment; (B-D) results after treatment with 10% SDS. (A) NDM-1 without pretreatment; (B) NDM-1; (C) KPC-2; (D) OXA-48; (E) OXA-48 and NDM-1.

A second, advanced protocol was developed for improved performance with NDM producing Enterobacteriaceae, Fig 2. The steps are the same as above, except that after 1 ml BC fluid was drawn from the BC bottles, 10 μl of a 0.5 M solution of zinc sulfate (ZnSO_4_) were added and the mixture was incubated for 15 min at 37°C in a dry heating block, shaking at 300/min. The remaining protocol remained unchanged, including hemolysis with 10% SDS, washing once or twice and lysis of the pellet with LY-A solution.

A detailed step-by-step protocol with additional pictures can be accessed at http://dx.doi.org/10.17504/protocols.io.rtrd6m6. [PROTOCOL DOI]

All tests were performed and read by the same technician to guarantee a standardized workup. When the result of the ICT was inconclusive, a second, blinded technician was consulted.

## Results

**Fig 3:**
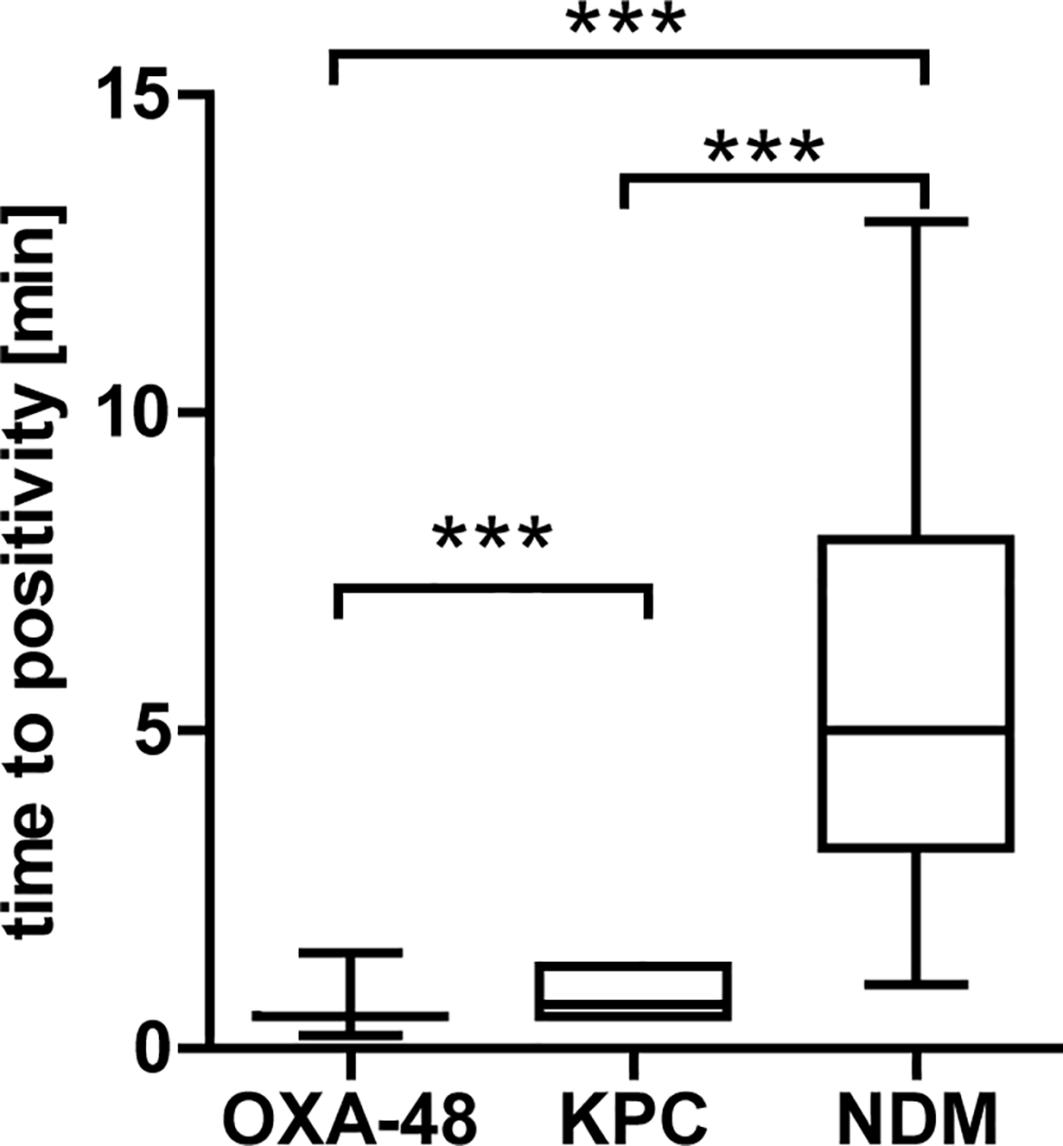
Time to positivity for OXA-48-like, KPC or NDM-type carbapenemases. *** indicates p<0.001.

In the first part of the evaluation, a protocol for the analysis of blood cultures with the ICT was developed. Therefore, blood culture bottles were spiked with five different CPE isolates and the blood culture fluid was processed either without any pretreatment or pretreated with four different hemolyzing agents. Additionally, different blood culture fluid volumes were assessed. Without pretreatment, only a low blood culture fluid volume (≤ 100 μL) could be used which subsequently resulted in a low sensitivity for NDM-producing isolates (Fig 2D and S2 Fig). Additionally, the red background of the erythrocytes compromised the reading of the bands of the ICT. When 1 mL of blood was lysed with 200 μL 10% SDS, the best results were achieved regarding intensity of the bands and time for the ICT to become positive (Fig 2 and Fig 3). Other hemolyzing agents (5% saponin, Triton-X or NH_4_Cl/KHCO_3_) or a lower blood volume resulted in either less complete hemolysis (S1 Fig), a lower intensity of the bands on the ICT or false negative results for NDM (data not shown). For the following evaluation, 126 CPE and 44 carbapenemase-negative isolates were tested after hemolysis with 10% SDS.

**Fig 4:**
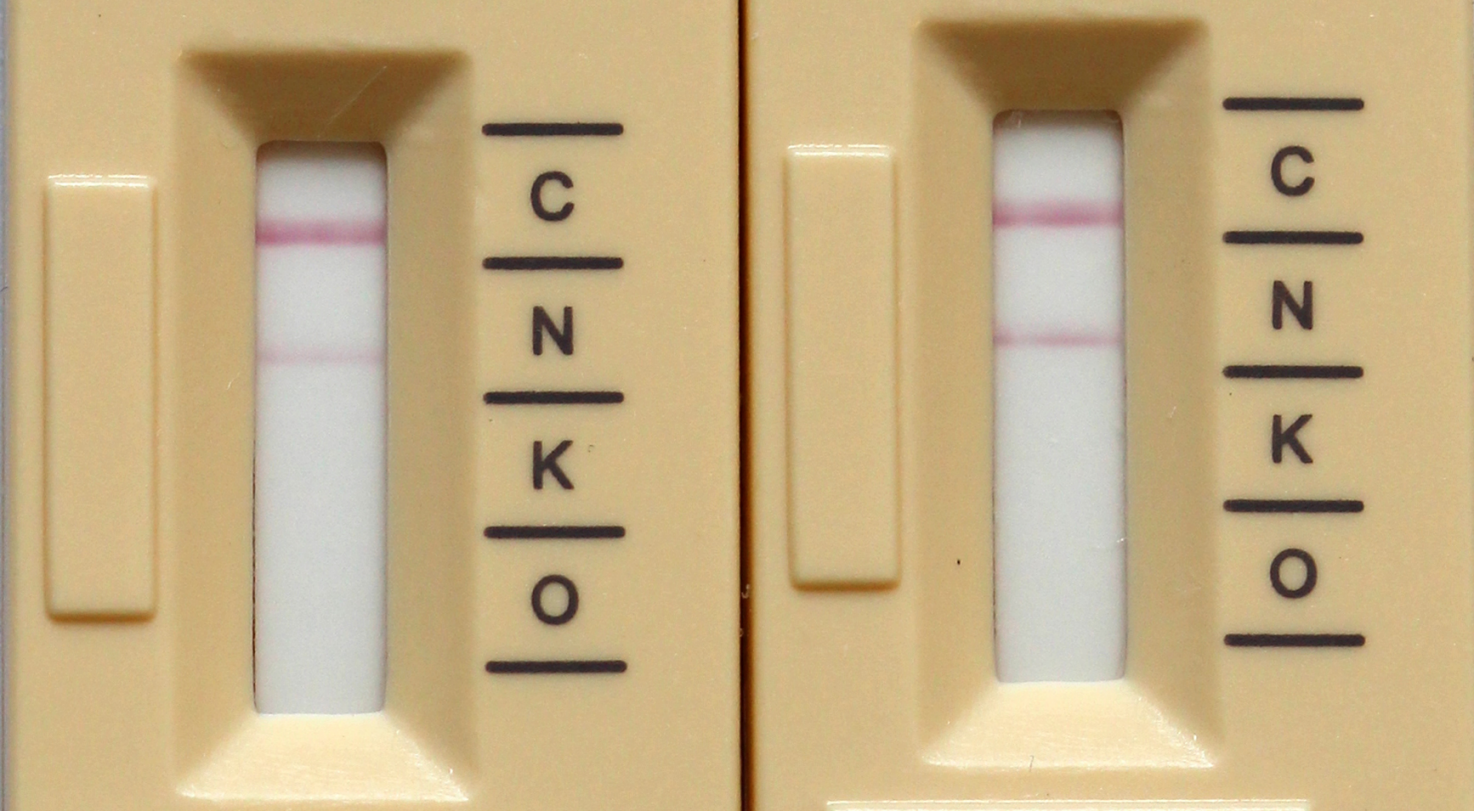
Effect of zinc on ICT. *K*. *pneumoniae* producing NDM-1 after processing with the short protocol (left) and advanced protocol (right). The intensity of the NDM band is increased when an incubation step with zinc sulfate is included.

Using 1 mL of blood culture fluid volume and after hemolysis with SDS, there was no red background and the bands on the ICT could be easily read (Fig 2 and S2 Fig). All carbapenemases were detected with the new protocol from BC and there were no false-positives, resulting in a sensitivity and specificity of 100% for OXA-48-like, KPC and NDM (Table 2). NDM isolates resulted in fainter bands on the ICT and became positive later (mean 5.8 ± 3.4 min) than isolates producing OXA-48-like (mean 0.54 ± 0.16 min, P<0.0001) or KPC carbapenemases (mean 0.79 ± 0.34 min, P<0.0001), Figs 1 and 2. In a few isolates, NDM bands of the ICT very faint and appeared only after the full 15 min reading time. For that reason, an advanced protocol was developed which includes an additional 15 min incubation step with zinc sulfate. Using this protocol, NDM isolates producing faint bands on the ICT could be read more easily (Fig 4) and the median time to positivity decreased from 5.8 min to 3 min, p = 0.003. The turn-around-time for the test was 20–30 min with the short protocol and 35–45 min with the advanced protocol.

**Table 2:** Sensitivity and specificity of the ICT for the detection of different carbapenemases directly from blood cultures (short protocol).

Carbapenemase	positive tests/ total isolates	Sensitivity (95% CI)	Specificity (95% CI)
**OXA-48-like**	79/79	100% (95.4–100%)	100% (96.1–100%)
OXA-48	54/54		
OXA-181	8/8		
OXA-244	2/2		
OXA-232	7/7		
OXA-162	7/7		
OXA-204	1/1		
**OXA-48-like/NDM**	3/3	100% (29.2–100%)	100% (97.8–100%)
OXA-232/NDM-1	1/1		
OXA-181/NDM-5	1/1		
OXA-48/NDM-1	1/1		
**KPC**	18/18	100% (81.5–100%)	100% (97.6–100%)
KPC-2	17/17		
KPC-3	1/1		
**NDM**	29/29	100% (88.1–100%)	100% (97.4–100%)
NDM-1	27/27		
NDM-5	1/1		
NDM-7	1/1		

## Discussion

This study demonstrates that with the newly developed protocols KPC, OXA-48-like and NDM carbapenemases can reliably be detected directly from positive blood cultures within 20–45 min. The sensitivity and specificity of the assay from blood cultures is equal to the sensitivity reported from solid media [8]. However, both the inoculum and hemolysis procedure were critical to obtain a sensitivity of 100%. ICTs from positive blood cultures have been reported for OXA-48 and recently for KPC using a low blood culture fluid volume (40 μL-100 μL) without hemolysis [14, 15]. Confirming the results of Wareham *et al*. [15], this procedure worked fine for OXA-48-like producing isolates, but not for NDM with frequent false-negatives, likely because of a lower sensitivity of the ICT for NDM. Without hemolysis, results of the ICT were more difficult to read because of the red background of the erythrocytes (Fig 2). Therefore, we used a volume of 1 mL to increase the bacterial inoculum and a hemolysis procedure to facilitate reading of the ICT. Using the short protocol, all carbapenemases could be detected and a sensitivity of 100% was achieved. However, NDM positive isolates resulted in fainter bands compared to KPC and OXA-48, which has also been observed for this ICT when NDM producing strains were harvested from solid media [9]. Therefore, as recommended by the manufacturer any band appearing on the ICT should be regarded as positive. For a better performance with NDM producing isolates, we additionally developed an advanced protocol including 15 min incubation with zinc sulfate. Zinc is a co-factor for metallo-β-lactamases and it has previously been demonstrated that it improves the detection of NDM with the ICT from solid media [9]. Using the advanced protocol, isolates with previously faint bands could be read more easily.

In a recent study NDM could not be reliably detected from blood cultures [16] using the same ICT as in the present study. However, both a smaller inoculum (100 μL or 500 μL) and no hemolysis procedure were used. Additionally, the ICT was assessed with blood culture bottles of a different manufacturer (bioMérieux BacT/Alert), which could have further influenced the results because of a different composition of the broth and resins. For that reason, we also assessed the performance of the ICT with the two protocols and BacT/ALERT FA Plus BC bottles. A total of 10 NDM-producing isolates was tested with the standard protocol and the advanced protocol and the results were compared to results of Bactec BC bottles, which were simultaneously inoculated. With both protocols 10/10 NDM isolates were detected, but bands were fainter with BactAlert compared to Bactec bottles. With the advanced protocol including zinc, ICT results for NDM could be read more easily also for BactAlert bottles.

Compared to the previous evaluations [9, 14, 15], in the present study more isolates with different carbapenemases were included and the multiplex ICT was evaluated for its use directly from positive blood cultures. The sensitivity and specificity was 100%, which was higher than previously reported when protocols without hemolysis and a smaller volume of blood culture fluid were used [14, 16].

Other methods have been reported for the detection of carbapenemases directly from positive blood cultures, e.g. the Carba NP test, MALDI-TOF hydrolysis assays or molecular methods, e.g. the Verigene assay [17–19]. Each approach has specific strengths and weaknesses regarding performance, associated costs and time to result. Compared to the Carba-NP and the MALDI-TOF hydrolysis assay, the advantage of the ICT-based protocol is the considerably shorter hands-on time and time to result. For the aforementioned techniques, additional broth cultures and longer incubation are necessary after a blood culture has become positive, requiring an additional three to five hours compared to 20–30 min for the ICT with the short and 35–45 min with the advanced protocol. With the Verigene assay, the time to result is longer (~2h) and the assay is considerably more expensive than the ICT. Furthermore, for both the MALDI-TOF hydrolysis assay and the Verigene assay, additional equipment and software is necessary. Most importantly, the sensitivity and specificity for NDM, OXA-48-like and KPC carbapenemases was higher (100%) with our ICT-based protocol than with either Carba NP, MALDI-TOF or Verigene assay [17–19]. However, with the ICT VIM and IMP type carbapenemases are currently not recognized but can be detected by the other methods.

In this proof of principle study, two new protocols for the detection of CPE from blood cultures using a multiplex ICT was developed and systematically assessed, resulting in the detection of the most important carbapenemases with 100% sensitivity and specificity. A rapid, 20–30 min protocol was developed and an advanced protocol for improved detection of NDM producing isolates. Especially in areas where NDM is frequent and for users of the BacT/Alert BC system, we recommend the advanced protocol, as reading is easier for NDM. In patients that are known to be colonized by KPC or OXA-48-like Enterobacteriaceae and where the exclusion of BSI with these carbapenemases is crucial, the short protocol may be used to further reduce handling time.

The study included a large number of molecularly characterized isolates with different carbapenemase variants. However, the study has some limitations. Since the prevalence of BSI with CPE at our institution is too low for a prospective evaluation, we had to use spiked blood cultures to include a sufficient number of isolates with different carbapenemase variants. To optimally simulate the conditions in bloodstream infections from patients, human blood was used instead of horse blood as in other studies [16]. However, our results should be verified in a routine microbiology laboratory setting with a sufficient number of patients with BSI caused by CPE. Additionally, we tested aerobic blood cultures from the two main manufacturers only (Becton Dickinson and bioMérieux). Results might not be identical when other blood culture media are used. In areas with a high prevalence of other carbapenemases not included in this multiplex ICT (e.g. VIM, IMP), the present ICT should be combined with other tests for the detection of these carbapenemases.

## Conclusions

The present study demonstrates that with the new protocols, OXA-48-like, KPC or NDM- carbapenemases from carbapenemase-producing Enterobacteriaceae can be reliably detected by the new multiplex ICT directly from positive blood culture bottles. With 20 to 45 min time to result, the new method is more rapid than other currently available assays and can be performed in any routine microbiology laboratory, as no additional equipment is required. Especially in regions with a high prevalence of OXA-48-like, NDM or KPC-producing Enterobacteriaceae and/or in patients known to be colonized by any of these CPE, our protocols may help to rapidly identify patients with CPE bloodstream infections and early optimize the management of patients with these difficult-to-treat infections. Further studies are needed to assess the performance of the ICT in routine diagnostics.

## Supporting information

S1 Fig Comparison of hemolysis with 10% SDS, Triton-X, saponin or NH_4_CL/KHCO_3_. The most complete hemolysis was achieved with 10% SDS.
(TIF)

S2 Fig Comparison of the effect of different volumes of blood culture fluid (BCF) on the intensity of the NDM band. *E*. *coli* NDM-1 was used as a test organism. On the right side, 100 μl BCF was used without prior hemolysis.
(TIF)

## References

[1] Falagas ME, Tansarli GS, Karageorgopoulos DE, Vardakas KZ. Deaths attributable to carbapenem-resistant Enterobacteriaceae infections. Emerg Infect Dis. 2014;20(7):1170–5. 10.3201/eid2007.121004 24959688 PMC4073868

[2] Nordmann P, Naas T, Poirel L. Global spread of Carbapenemase-producing Enterobacteriaceae. Emerg Infect Dis. 2011;17(10):1791–8. 10.3201/eid1710.110655 22000347 PMC3310682

[3] Koroska F, Göttig S, Kaase M, Steinmann J, Gatermann S, Sommer J, et al Comparison of Phenotypic Tests and an Immunochromatographic Assay and Development of a New Algorithm for Detection of OXA-48-like Carbapenemases. Journal of clinical microbiology. 2017;55(3):877–83. 10.1128/JCM.01929-16 28031433 PMC5328455

[4] Giske CG, Martinez-Martinez L, Canton R, Stefani S, Skov R, Glupczynski Y, et al EUCAST guidelines for detection of resistance mechanisms and specific resistances of clinical and/or epidemiological importance. 07/2017 ed2017. p. 1–43.

[5] Glupczynski Y, Jousset A, Evrard S, Bonnin RA, Huang TD, Dortet L, et al Prospective evaluation of the OKN K-SeT assay, a new multiplex immunochromatographic test for the rapid detection of OXA-48-like, KPC and NDM carbapenemases. The Journal of antimicrobial chemotherapy. 2017;72(7):1955–60. 10.1093/jac/dkx089 28369469 PMC5890672

[6] Meunier D, Vickers A, Pike R, Hill RL, Woodford N, Hopkins KL. Evaluation of the K-SeT R.E.S.I.S.T. immunochromatographic assay for the rapid detection of KPC and OXA-48-like carbapenemases. The Journal of antimicrobial chemotherapy. 2016;71(8):2357–9. 10.1093/jac/dkw113 27118775

[7] Rubio E, Zboromyrska Y, Pitart C, Campo I, Alejo-Cancho I, Fasanella A, et al Evaluation of a rapid immunochromatographic test for the detection of OXA-48 carbapenemase. Diagnostic Microbiology and Infectious Disease. 2017;87(3):266–7. 10.1016/j.diagmicrobio.2016.12.001 27988171

[8] Wareham DW, Abdul Momin MH. Rapid Detection of Carbapenemases in Enterobacteriaceae: Evaluation of the Resist-3 O.K.N. (OXA-48, KPC, NDM) Lateral Flow Multiplexed Assay. Journal of clinical microbiology. 2017;55(4):1223–5. 10.1128/JCM.02471-16 28151407 PMC5377851

[9] Saleh A, Gottig S, Hamprecht AG. Multiplex Immunochromatographic Detection of OXA-48, KPC, and NDM Carbapenemases: Impact of Inoculum, Antibiotics, and Agar. Journal of clinical microbiology. 2018;56(5). 10.1128/JCM.00050-18 PMC5925695 29444829

[10] Hamprecht A, Rohde AM, Behnke M, Feihl S, Gastmeier P, Gebhardt F, et al Colonization with third-generation cephalosporin-resistant Enterobacteriaceae on hospital admission: prevalence and risk factors. The Journal of antimicrobial chemotherapy. 2016;71(10):2957–63. 10.1093/jac/dkw216 27317445

[11] Jazmati N, Hein R, Hamprecht A. Use of an Enrichment Broth Improves Detection of Extended-Spectrum-Beta-Lactamase-Producing Enterobacteriaceae in Clinical Stool Samples. Journal of clinical microbiology. 2016;54(2):467–70. 10.1128/JCM.02926-15 26607984 PMC4733169

[12] Göttig S, Hamprecht AG, Christ S, Kempf VA, Wichelhaus TA. Detection of NDM-7 in Germany, a new variant of the New Delhi metallo-beta-lactamase with increased carbapenemase activity. The Journal of antimicrobial chemotherapy. 2013;68(8):1737–40. 10.1093/jac/dkt088 23557929

[13] Gruber TM, Gottig S, Mark L, Christ S, Kempf VA, Wichelhaus TA, et al Pathogenicity of pan-drug-resistant Serratia marcescens harbouring blaNDM-1. The Journal of antimicrobial chemotherapy. 2015;70(4):1026–30. 10.1093/jac/dku482 25468904

[14] Riccobono E, Antonelli A, Pecile P, Bogaerts P, D'Andrea MM, Rossolini GM. Evaluation of the KPC K-SeT(R) immunochromatographic assay for the rapid detection of KPC carbapenemase producers from positive blood cultures. The Journal of antimicrobial chemotherapy. 2018;73(2):539–40. 10.1093/jac/dkx406 29126269

[15] Wareham DW, Shah R, Betts JW, Phee LM, Momin MH. Evaluation of an Immunochromatographic Lateral Flow Assay (OXA-48 K-SeT) for Rapid Detection of OXA-48-Like Carbapenemases in Enterobacteriaceae. Journal of clinical microbiology. 2016;54(2):471–3. 10.1128/JCM.02900-15 26607983 PMC4733200

[16] Wareham DW, Phee LM, Abdul Momin MHF. Direct detection of carbapenem resistance determinants in clinical specimens using immunochromatographic lateral flow devices. The Journal of antimicrobial chemotherapy. 2018. 10.1093/jac/dky095 29579199

[17] Dortet L, Brechard L, Poirel L, Nordmann P. Rapid detection of carbapenemase-producing Enterobacteriaceae from blood cultures. Clinical microbiology and infection: the official publication of the European Society of Clinical Microbiology and Infectious Diseases. 2014;20(4):340–4. 10.1111/1469-0691.12318 23889766

[18] Fernández J, Rodríguez-Lucas C, Fernández-Suárez J, Vazquez F, Rodicio MR. Identification of Enterobacteriaceae and detection of carbapenemases from positive blood cultures by combination of MALDI-TOF MS and Carba NP performed after four hour subculture in Mueller Hinton. Journal of Microbiological Methods. 2016;129:133–5. 10.1016/j.mimet.2016.08.014 27546715

[19] Ledeboer NA, Lopansri BK, Dhiman N, Cavagnolo R, Carroll KC, Granato P, et al Identification of Gram-Negative Bacteria and Genetic Resistance Determinants from Positive Blood Culture Broths by Use of the Verigene Gram-Negative Blood Culture Multiplex Microarray-Based Molecular Assay. Journal of clinical microbiology. 2015;53(8):2460–72. 10.1128/JCM.00581-15 25994165 PMC4508435

