# Supplementary figures and images for: Rapid detection of NDM, KPC and OXA-48 carbapenemases directly from positive blood cultures using a new multiplex immunochromatographic assay
Source: PLoS One. 2018 Sep 14;13(9):e0204157. doi: 10.1371/journal.pone.0204157 (PMC6138386; doi:10.1371/journal.pone.0204157)

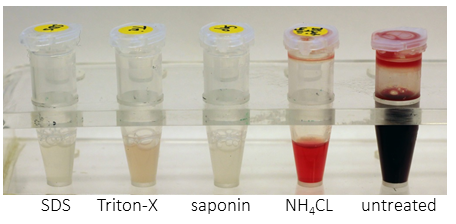

Supplement: S1 Fig — The most complete hemolysis was achieved with 10% SDS. (TIF) [file pone.0204157.s001.tif]

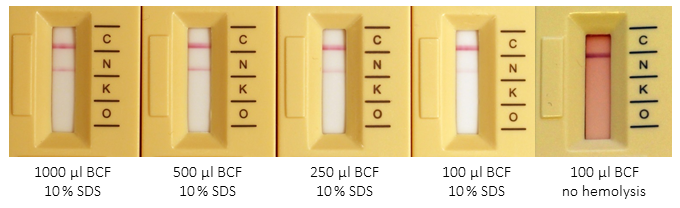

Supplement: S2 Fig — E. coli NDM-1 was used as a test organism. On the right side, 100 μl BCF was used without prior hemolysis. (TIF) [file pone.0204157.s002.tif]
